# The Keystone Veterinary Medical Association

**Published:** 1890-04

**Authors:** W. H. Ridge

**Affiliations:** Secretary


*The Keystone Veterinary Medical Association* held its monthly meeting at the College of Physicians, Philadelphia, March 1st, 1890. Dr. Hoskins, vice-president, presided. Owing to its being a stormy night only ten members were present: Drs. W. B. Miller, Goentner, Hoskins, T. B. Raynor, J. B. Raynor, Kooker, Bridge, Ridge, Lusson and Magill. The minutes of the February meeting were read and approved.

The Legislative Commitee reported 752 veterinary surgeons in this State, of which 157 are graduates; there are 218 illegal registries, of which 82 are post-registries. There has been no trial of any illegal practitioner, but there have been several arrests. The committee will test the strength of the law. It was moved that the amendment of the by-laws be laid over until next month.

Dr. J. B. Raynor asked if anyone residing in an adjoining State could practice in this State without a license. There was a diversity of opinion on this subject.

The Committee on Essays reported. For May, William B. E. Miller, D.V.S. Subject, "Castration."

Dr. J. B. Raynor reported a case of dystocia. Subject, a cow; had been in labor three days, lying on side; tympanitic; vagina dry; on exploration he found fœtus dead; posterior presentation, lumbo-pubic position, with

[1] Owing to want of space this paper must be held for our next issue.—ED.]

hind legs not presented; he performed embryotomy and used stimulating treatment, with injections of starch water into uterus. The cow recovered.

Dr. Goentner reported a case of rabies. He was called to see a gelding in January, which had been ailing since the day before. He could get no history at the time of first visit. The animal stood tucked up and had a pinched look. Its temperature was 104° F. It was very restless, and had a slight tremor of the flanks. The eyes stared with a wild look, the ears moved backward and forward. He could not make a diagnosis on the few symptoms, but was suspicious of the disease, and cautioned the owner. The next day he was sent for again, and on examining the animal found that it had pawed a deep hole in the ground with its off fore foot. The ears had the same motion, the twitching of the muscles was more aggravated, the abdomen was tucked up. On starting to go into the box containing the animal, it reared and struck at the doctor. The horse caught hold of a piece of the partition with his teeth, breaking or tearing out all of his incisor teeth at the first bite; he went on furiously for a few minutes and then became more calm. When offered some water in a bucket, after swallowing a few mouthfuls, it produced such spasms of the pharynx and œsophagus that the animal could not swallow. The doctor made a diagnosis of rabies. He found out afterward that the horse had been bitten by a dog a few weeks before. He thought it important to be able to diagnose rabies in a horse at first glance, and that, too, at a few rods off.

Dr. Thomas B. Raynor cited several cases. Dr. William B. E. Miller told of a horse in Gloucester County, N. J., that he saw, which acted much like the one Dr. Goentner reported. Dr. Hoskins reported a case of a pet dog that he attended a few weeks ago. The dog seemed fidgety, ran up-stairs and down, would lap a little milk every few minutes, and was unusually affectionate. The dog finally made the peculiar howl so characteristic of rabies, and the diagnosis was made at once. The dog was chained in a room. The next day they found the paper torn from the walls, the carpet torn into shreds, and the dog was wild with fury, leaving no doubt as to the diagnosis.

By motion adjourned.

W. H. RIDGE, *Secretary*.

